# Patent license prediction using deep survival analysis: A comparative study

**DOI:** 10.1371/journal.pone.0355826

**Published:** 2026-09-09

**Authors:** Takao Arai, Hiroyasu Inoue

**Affiliations:** 1 Graduate School of Information Science, University of Hyogo, Kobe, Hyogo, Japan; 2 Center for Computational Science, RIKEN, Kobe, Hyogo, Japan; State University of New York at Oswego, UNITED STATES OF AMERICA

## Abstract

**Objective:**

We evaluate deep survival analysis frameworks for patent licensing prediction, systematically comparing cure rate models against standard approaches and quantifying the benefit of temporal modeling over static classification to address unresolved methodological questions following recent applications of neural survival models to patent contexts.

**Methods:**

We conducted experimental validation on 624,129 United States Patent and Trademark Office (USPTO) patents (2016–2017), comparing classical Cox regression, deep neural network baseline, DeepSurv (neural Cox proportional hazards), and Cox cure rate models. Performance was evaluated using Recall@K metrics across 1-year, 3-year, and 5-year prediction horizons, with statistical significance assessed through patent-level bootstrap resampling (*B* = 1,000).

**Results:**

All deep learning approaches achieve high performance (Recall@10% 89.8–91.9%), substantially outperforming classical Cox regression (70.6–72.3%). Among neural architectures, DeepSurv—a standard neural Cox proportional hazards model—offers the optimal balance of simplicity and performance. More complex cure rate extensions provided no clear additional benefit over standard DeepSurv within the scope of this study: although bootstrap resampling detected a small but statistically significant difference (0.70 percentage points), this gap is practically negligible for patent screening, suggesting that explicit population heterogeneity modeling offers limited practical gains when deep feature learning is employed. Survival modeling provides statistically detectable but modest gains over static binary classification (Recall@10% differences of 0.5–1.6 percentage points), indicating that the primary value stems from capturing complex patent characteristics rather than explicit temporal modeling.

**Conclusion:**

This study provides, to the best of our knowledge, the first systematic evaluation of cure rate models and temporal modeling benefits for patent licensing prediction, demonstrating that architectural simplicity (DeepSurv) achieves near-optimal performance (Recall@10% 91%) when deep feature learning is prioritized. The findings offer actionable guidance for patent portfolio management: invest in feature learning infrastructure rather than architectural sophistication.

## Introduction

Accurate early identification of valuable patents enables firms, universities, and investors to focus on costly expert reviews on a small, high-value subset of large portfolios and informs evidence-based intellectual property (IP) decision making. The measurement of patent value presents fundamental challenges due to the multifaceted nature of intellectual property. Existing approaches rely on various proxy indicators, each capturing different aspects of patent value. Forward citations serve as indicators of technological impact [1,2], patent maintenance periods reflect commercial commitment [3], and citation timing patterns reveal knowledge diffusion dynamics [4]. However, these proxies tend to emphasize technological aspects more than direct commercial value indicators. Patent licensing, on the contrary, represents direct market validation: a transaction in which parties explicitly assign monetary value to intellectual property rights. This distinction makes licensing prediction particularly valuable for IP management and investment decisions [5].

The temporal dimension is critical in the assessment of patent value. Research shows that timing significantly impacts value, from the technological influence indicated by early-forward citations to maintenance decisions that evolve throughout the lifecycle of a patent based on changing commercial prospects [3,4]. Recent advances in patent portfolio valuation, such as the Patent Portfolio Value Index (PPVI), build on this by integrating temporal factors into a broader framework. PPVI synthesizes quantitative data, including time-sensitive metrics such as patent age and citation frequency, with strategic qualitative assessments to provide a more holistic evaluation [6].

However, patent licensing presents unique temporal challenges that distinguish it from other patent value indicators. Unlike forward citations or maintenance decisions that follow relatively predictable patterns, licensing timing involves complex strategic considerations. Kim et al. [7] demonstrate that licensing timing depends on intricate trade-offs between technological capabilities, patent protection stages, and appropriation concerns, factors that create deliberate delays even with the most suitable licensees. This strategic complexity means that licensing events can occur at any point throughout the lifetime of a patent, with commercial value potentially emerging years after initial filing and being influenced by evolving market conditions, competitive landscapes, and technological developments.

Survival analysis provides a natural framework for modeling time-to-event data with censoring. Classical approaches such as Cox proportional hazards regression have been widely applied to model hazard functions using hand-crafted features, establishing baseline methodologies for temporal prediction. Recent advances in deep learning have extended these frameworks in two directions: standard deep survival models such as DeepSurv [8] that learn complex feature representations while maintaining proportional hazards assumptions, and cure rate models [9,10] that explicitly account for population heterogeneity by distinguishing subjects who will eventually experience events from those who never will. These methodological developments, combined with traditional binary classification approaches commonly used in patent prediction [11,12], offer complementary perspectives on licensing prediction that warrant systematic comparison.

Predicting future licensing presents fundamental methodological challenges arising from these temporal complexities. Most license contracts are private, with only a fraction publicly disclosed, creating scenarios where licensing prediction must operate under incomplete information. Additionally, licensing is inherently a time-to-event process, where newer patents have had less time for licensing transactions to occur and be reported. Treating licensing as a static binary classification problem ignores both right-censoring from observation windows and the temporal dynamics of licensing decisions, potentially leading to biased models and misleading evaluations.

Prior work has approached patent licensing prediction primarily through static machine learning classification [2,11–14], treating licensing as a binary outcome without modeling temporal aspects. Although survival analysis has been applied to understand licensing dynamics in healthcare patents [15], integrating topic modeling with Cox models to assess commercialization likelihood, this approach has focused primarily on identifying causal factors rather than developing predictive models for large-scale patent portfolios. Moreover, traditional Cox models rely on proportional hazards assumptions and cannot adequately capture the complex time-varying hazards and nonlinear temporal dependencies inherent in patent licensing decisions.

To address these limitations, deep learning approaches to survival analysis have shown promise in capturing time-varying hazards and modeling time-to-event phenomena. Recent applications to patent contexts have demonstrated the viability of neural survival models [16,17], yet critical methodological questions remain unresolved. First, patent licensing may involve inherent population heterogeneity: some patents may be fundamentally unlicensable due to technical obsolescence, narrow claim scope, or market misalignment, while others face only delayed licensing events. Cure rate models, developed to distinguish such permanent non-susceptibility from censored observations [9,10], offer a natural framework for this setting—but their value relative to standard deep survival models remains empirically unvalidated in patent contexts. Second, the quantifiable benefit of temporal modeling over static classification approaches has not been systematically evaluated. Answering these questions is essential for practical deployment, where model complexity must be justified by measurable performance gains.

This study applies deep survival analysis to systematically address these unresolved questions in patent licensing prediction, evaluating multiple frameworks that handle the temporal dimension and right-censoring inherent in patent datasets. We compare four modeling approaches representing key methodological variations: (1) classical Cox regression with hand-crafted features, (2) deep neural network for static binary classification (representing the prior static prediction paradigm), (3) DeepSurv for neural Cox proportional hazards modeling, and (4) Cox cure rate model to account for potential population heterogeneity where some patents may never be licensed. These four models are chosen to form a controlled comparison: the three survival models share a common continuous-time, Cox-based proportional-hazards foundation and differ only in feature representation and the presence of an explicit cure fraction, while the deep neural network serves as a static binary-classification reference that isolates the value of temporal modeling. The rationale for this design, the resulting scope of our comparison, and our reasons for deferring survival paradigms built on different foundations to future work are detailed in the Model Architectures section.

The primary contributions of this research are threefold. First, we provide, to the best of our knowledge, the first systematic evaluation of continuous-time, Cox-based deep survival analysis frameworks for large-scale patent licensing prediction, properly handling right-censoring and temporal dynamics that existing machine learning approaches ignore. Second, our comparative analysis on 624,129 United States Patent and Trademark Office (USPTO) patents demonstrates that all deep learning approaches achieve high performance (Recall@10% 89.8–91.9%), representing substantial improvement over classical Cox regression (70.6–72.3%) and random baseline (10%). Third, our findings indicate that, within this dataset and experimental setting, cure rate modeling provides limited additional benefit over standard DeepSurv, offering practical guidance that performance gains primarily stem from deep feature learning. These results establish deep survival analysis as a viable framework for patent portfolio management while providing actionable insights for model selection in practice.

## Related works

Research on patent licensing prediction spans multiple domains, from econometric studies of licensing determinants to machine learning approaches for patent value assessment. Our work bridges these areas by developing a deep survival analysis framework specifically designed for patent licensing contexts with temporal modeling capabilities.

### Patent value assessment

The challenge of quantifying patent value has necessitated diverse measurement approaches, each targeting different outcome variables that serve as proxies for intellectual property value. Forward citation counts represent the most established target variable [1,18,19], though they primarily capture technological rather than commercial significance [20]. Alternative approaches target patent maintenance decisions [3], network centrality measures [2], and quality scores [14]. These diverse targets reflect fundamental trade-offs: citation-based outcomes may reflect academic rather than commercial interest, maintenance decisions depend on firm-specific strategies, and network centrality emphasizes positional importance rather than market potential [5]. Patent licensing outcomes, in contrast, represent explicit market transactions in which parties negotiate monetary value for intellectual property rights, providing direct indicators of commercial value that distinguish them from these indirect proxies.

### Temporal dynamics in patent analytics

The temporal dimension has gained increasing recognition in patent value assessment. Survival analysis of patent maintenance reveals that value indicators evolve over time, with family size emerging as the strongest early predictor while forward citations show delayed effects [3]. Recent advances in machine learning-based survival analysis have enabled more accurate predictions of patent lifespan by incorporating multidimensional characteristics including technical, legal, market, patentee features, and textual semantic features [21]. Citation timing analysis demonstrates that patents with shorter technology cycle times, larger patent families, and higher claim counts receive citations more quickly [4].

However, licensing timing differs fundamentally from citation or maintenance patterns. Research on university technology licensing reveals complex strategic considerations, showing that licensees with strong technological capabilities tend to pursue early licensing before patent rights are fully established, though this tendency is moderated by appropriation concerns when significant technological overlap exists [7]. Survival analysis using Cox proportional hazards models has been applied to healthcare patent licensing prediction, integrating topic modeling with expert judgment [15]. Unlike citations that reflect technological building upon prior work or maintenance decisions at predetermined intervals, licensing events exhibit heterogeneous timing patterns influenced by technology readiness levels, market development cycles, commercial viability assessments, and competitive dynamics emerging at various points during a patent’s lifetime. This distinct temporal complexity suggests that licensing prediction requires specialized modeling approaches that can capture the unique timing characteristics of commercial technology transfer events.

### Patent licensing prediction models

The PatVal-EU study [22] provided empirical evidence showing that about 11% of patents were licensed, with an additional 7% of patent owners willing but unable to license due to transaction costs, demonstrating that factors affecting willingness to license differ from those affecting actual licensing probability. Predictive modeling approaches have evolved from early work using neural networks for patent transferability [13] to sophisticated machine learning approaches integrating patent-based features [11] and Graph Convolutional Networks modeling licensing relationships [12]. Recent research has emphasized patent scope measures, with citation network analysis revealing that dynamic characteristics, particularly inward degree centrality, are significant factors in transfer prediction [23]. Transformer-based approaches using BERT embeddings [14] and domain adaptation techniques [24] have further advanced the field. However, these approaches treat patent transfer as a classification problem without accounting for censoring effects, where non-transferred patents during observation periods may eventually transfer in the future, potentially leading to biased predictions that conflate truly non-transferable patents with those whose transfer events have not yet been observed.

### Deep survival analysis

Deep survival models, developed primarily in medical and social science domains, extend classical survival analysis with neural networks, enabling analysis of high-dimensional feature spaces and nonlinear relationships while properly handling right-censored data. Established frameworks include DeepSurv [8], which implements neural Cox proportional hazards models, and DeepHit [25], which models survival distributions through discrete-time competing risks frameworks. Despite their success in medical and social applications, deep survival models have seen limited adoption in patent analytics. Marusaki et al. [16] applied them to patent maintenance duration prediction, and Rhee et al. [17] recently demonstrated DeepSurv’s superiority over Random Survival Forests and classical Cox models on Korean patent transaction data, establishing neural survival models as viable for patent transaction timing prediction.

Standard survival models assume a single homogeneous population where all subjects will eventually experience the event of interest. Classical cure models [9] address population heterogeneity by explicitly modeling both susceptible and non-susceptible subgroups, with recent deep learning extensions demonstrating feasibility for recidivism prediction [10]. In patent licensing contexts, this framework may be particularly relevant as many patents could be inherently unlicensable due to technical obsolescence, narrow claim scope, or market misalignment. However, the relative merits of standard deep survival models versus cure rate extensions for patent licensing prediction remain unexplored. Moreover, prior work has not systematically compared survival modeling against static binary classification approaches to quantify the value of explicit temporal modeling. This study addresses these gaps by providing, to the best of our knowledge, the first systematic comparison of these approaches—including cure rate models and static classification baselines—on large-scale patent licensing data characterized by extreme class imbalance and heavy censoring.

## Methods

### Data acquisition

**Fig 1:**
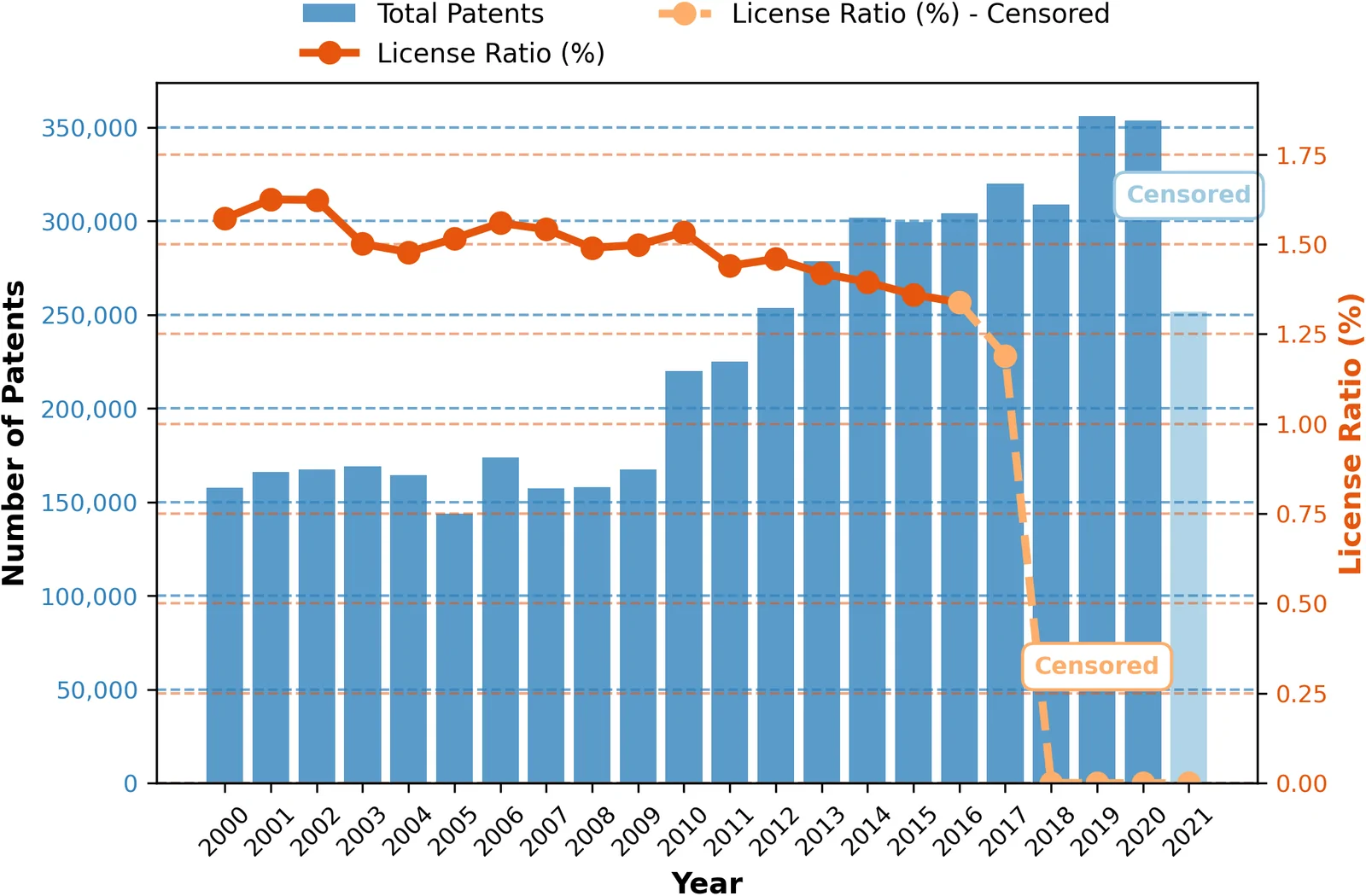
Annual patent volume and licensing ratio trends from 2000–2021. The dual-axis chart shows the total number of patents (blue bars, left y-axis) and corresponding license ratio percentage (orange line, right y-axis) over a 21-year period.

We retrieved the complete bibliographic, textual and citation record of every utility patent published in the United States between 2000 and 2021 from the Google Patents Public Data tables [26]. Licensing data was obtained from the USPTO Patent Assignment Dataset [27]. Because only two licensing events were found in this dataset after 2018, we restricted the licensed sample to USPTO events dated up to the end of 2017 (Fig 1).

Patent volume shows consistent growth from approximately 158,000 patents in 2000 to peak levels exceeding 355,000 patents in 2019–2020. The apparent decline to around 251,000 patents in 2021 reflects data truncation due to incomplete observation of the full year 2021 (the observation period ended on 28 September 2021). Similarly, the license ratio shows a decreasing trend from 1.5% in 2000 to approximately 0.0% by 2018–2021. This declining pattern in license ratios for more recent years reflects data censoring effects, as there is typically a time lag between patent publication and subsequent licensing activities. Both the reduced patent count in 2021 and the consistently declining licensing ratios in recent years are artifacts of the observation period ending partway through 2021, rather than representing actual decreases in patenting activity or licensing rates.

For each normalized publication number, the dataset contains:

- Application date, publication date, document type and claim count;
- The full abstract;
- The ordered concatenation of all claim sentences;
- The complete set of current Cooperative Patent Classification (CPC) group identifiers;
- Disambiguated assignee names (organizations or inventors) recorded at the time of patent grant, which remain fixed and do not reflect subsequent ownership transfers;
- USPTO-internal backward citations; and
- Voluntarily filed license information (licensee name and license date).

For USPTO licensing data, we use the execution date of the licensing agreement as the licensing event time. Survival time is calculated as the number of days from the patent application date to the execution date. In cases where the execution date preceded the application date (1,468 cases, 2.65% of licensed patents), we set the survival time to zero during model training to accommodate the non-negative survival time requirement. Among licensed patents, the median time-to-licensing was 1.6 years (IQR: 0.3–4.7 years), with 42.2% licensed within 1 year, 64.3% within 3 years, and 76.6% within 5 years of application. This concentration of early licensing events is consistent with prior findings by Graham et al. [27], who observed that many ownership changes occur within 3 years of grant.

The USPTO Patent Assignment Dataset captures licensing transactions through voluntary disclosure filings submitted by patent holders and licensees. Parties are not legally required to record licensing agreements with the USPTO, although they may choose to do so to establish public notice of their rights or for other strategic reasons. Consequently, the dataset represents a subset of actual licensing activity, with many licensing transactions remaining unrecorded. This voluntary nature means that patents labeled as unlicensed in our dataset may include cases where licensing occurred but was not disclosed, creating an incomplete but valuable record of observable market transactions in intellectual property rights.

When multiple license events were recorded for a single patent, we used the earliest license date as the event time for survival analysis. This approach treats the first observed licensing transaction as the primary event of interest, consistent with the economic interpretation that initial licensing represents the patent’s entry into commercial use. This methodology ensures that each patent contributes exactly one survival time to the analysis, maintaining the standard assumptions of survival modeling while capturing the most temporally proximate licensing event to patent publication. All underlying data are publicly available and do not contain personally identifiable information.

We employ a temporal split strategy to ensure a realistic evaluation of the out-of-sample. Given the large sample size (*n* = 2,903,422 for training; *n* = 299,382 for validation), cross-validation was not necessary for the estimation of stable parameters. Our approach mirrors real-world deployment scenarios in which models trained on data up to a certain point are used to make predictions about subsequent periods.

Patents were ordered chronologically and partitioned as follows.

- Training period (2000–2014). All patents published between 2000 and 2014 (*n* = 2,903,422; licensed = 43,502, 1.50%) constituted the development pool. This dataset was used for weight updates.
- Validation period (2015). All patents published in 2015 (*n* = 299,382; licensed = 4,070, 1.36%) constituted the validation set. This dataset was crucial for parameter tuning and model selection.
- Test period (2016–2017). All patents published between 2016 and 2017 (*n* = 624,129; licensed = 7,872, 1.26%) were held out ex ante and used for USPTO-based evaluation.

This strictly time-stratified protocol mirrors real-world deployment, preventing information leakage from future documents into model training.

### Model architectures

We evaluated four modeling approaches representing key methodological variations in survival analysis and patent prediction: (1) classical Cox regression baseline, (2) deep neural network for static binary classification, (3) DeepSurv implementing neural Cox proportional hazards, and (4) Cox cure rate model.

These four models are designed as a controlled, ablation-style comparison. The three survival models—the classical Cox baseline, DeepSurv, and the Cox cure rate model—share a common continuous-time, Cox-based proportional-hazards foundation and differ only in individual modeling components, namely feature representation (hand-crafted versus learned) and the presence of an explicit cure fraction; the deep neural network is included as a static binary-classification reference, so that its contrast with the survival models isolates the value of temporal modeling. Restricting the comparison to this shared foundation is what allows performance differences to be attributed to specific components: survival paradigms built on fundamentally different assumptions—such as DeepHit [25], which uses a discrete-time competing-risks formulation, and Random Survival Forests [28], which is tree-based and does not assume proportional hazards—would change the modeling foundation itself and confound this attribution, and are therefore outside the present scope and deferred to future work.

By design, each approach is also paired with the feature construction under which it is conventionally deployed: the classical Cox model uses interpretable hand-crafted features, whereas the deep models use learned embedding representations. This is an intentional choice that represents each paradigm in its standard, realistic form rather than forcing an artificial common input; in particular, supplying high-dimensional dense text embeddings directly to a linear Cox model would not reflect how such a model is used in practice. As a result, the comparison between the classical Cox baseline and the deep models is a contrast between entire modeling pipelines (method together with its typical feature representation), not between architectures in isolation, whereas the three deep models (DNN, DeepSurv, and Cox Cure) share an identical feature encoder, so comparisons among them isolate the effect of the temporal and cure-modeling components.

The classical Cox baseline implements standard Cox proportional hazards with linear predictor $\beta^{\prime }X$ where *X* represents a 999-dimensional hand-crafted feature vector comprising: scaled claim count (1), scaled backward citation count (1), 768-dimensional text embeddings from BERT-for-Patents [29], binary indicators for all 129 CPC codes, and binary indicators for the top 100 most frequent assignees. These features are processed directly without neural encoding.

Neural models (DNN, DeepSurv, Cox Cure) employ identical feature encoders processing 1042-dimensional input vectors comprising: scaled claim count (1), scaled backward citation count (1), 768-dimensional text embeddings from BERT-for-Patents [29], 16-dimensional mean-pooled CPC classification embeddings (vocabulary size 129), and 256-dimensional mean-pooled patent assignee embeddings (vocabulary size 207,499). The shared encoder consists of fully connected layers (1042→1024→512) with ReLU activations and dropout (0.30) after each layer, feeding into model-specific prediction heads.

The DNN model uses a prediction head consisting of fully connected layers (512→256→128→1) with ReLU activations, dropout (0.30), and sigmoid output. Three separate instances are trained independently for 1-year, 3-year, and 5-year prediction horizons.

DeepSurv implements neural Cox proportional hazards with an identical prediction head architecture but linear output representing log hazard ratio $\beta^{\prime }X$, with a single model predicting across all time horizons.

**Fig 2:**
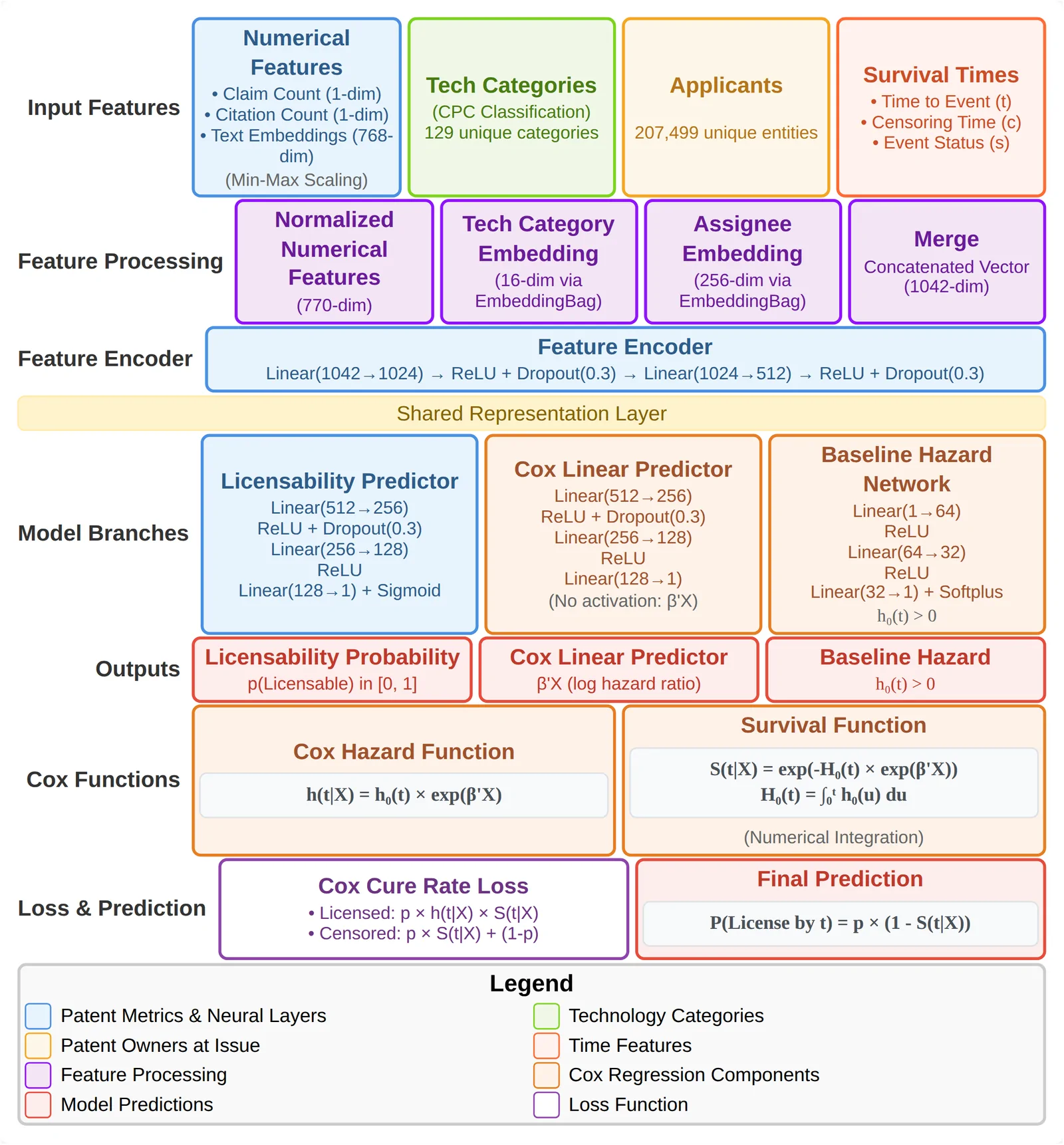
Semi-parametric Cox cure rate model architecture. The model processes patent features through a shared encoder (1042→1024→512 dimensions) that feeds into three specialized components: a licensability predictor outputting probability *p*, a Cox linear predictor outputting log hazard ratio $\beta^{\prime }X$, and a learnable baseline hazard function *h*_0_(*t*). The final licensing probability combines these components as P(License by t)=p×(1−S(t|X)), where $S(t|X)=\exp(-H_{0}(t)\times \exp(\beta^{\prime }X))$ is the survival function with Cox proportional hazards framework.

The Cox cure rate model extends DeepSurv with two parallel prediction heads from the shared encoder (Fig 2). The licensability predictor uses layers (512→256→128→1) with ReLU activations, dropout (0.30), and sigmoid output, producing probability p∈[0,1] that a patent is ever licensable. The Cox linear predictor uses identical architecture to DeepSurv, producing log hazard ratio $\beta^{\prime }X$ for licensable patents. An additional learnable baseline hazard network (1→64→32→1) with ReLU activations and Softplus output models *h*_0_(*t*).

The final licensing probability at time *t* combines both components:


$$\begin{array}{c} P(\text{License by }t)=p\times (1-S(t|X)) \end{array}$$ (1)

where the survival function follows the Cox proportional hazards framework:


$$\begin{array}{c} S(t|X)=\exp\left(-H_{0}(t)\times \exp(\beta^{\prime }X)\right) \end{array}$$ (2)

and $H_{0}(t)=\int_{0}^{t}h_{0}(u)du$ is the cumulative baseline hazard.

### Training procedure

Neural models employed Adam optimizer (learning rate 10^−4^, weight decay 10^−4^, batch size 256) with early stopping (patience 10 epochs) and maximum 50 training epochs. Cox Cure required additional optimization techniques for training stability: learning rate scheduling (ReduceLROnPlateau with factor 0.5, patience 3), gradient clipping (maximum norm 5.0), and 3-epoch warm-up with linear learning rate scaling from $\frac{1}{3}$ to full rate. DNN and DeepSurv trained stably without these techniques. Classical Cox baseline used LBFGS optimization with L2 regularization (α=0.1, tolerance 10^−9^, maximum 100 iterations).

DNN employed binary cross-entropy loss with class weights inversely proportional to class frequencies:


$$\begin{array}{c} {ℒ}_{\text{DNN}}=-\frac{1}{n}\sum_{i=1}^{n}w_{i}\left[y_{i}\log{\hat{y}}_{i}+(1-y_{i})\log(1-{\hat{y}}_{i})\right] \end{array}$$ (3)

where $w_{i}$ addresses extreme class imbalance.

DeepSurv implemented the Cox negative log-partial likelihood:


$$\begin{array}{c} {ℒ}_{\text{DeepSurv}}=-\sum_{i\in \text{events}}\left[\beta^{\prime }X_{i}-\log\sum_{j\in R(t_{i})}\exp(\beta^{\prime }X_{j})\right] \end{array}$$ (4)

where $R(t_{i})$ denotes the risk set at time $t_{i}$.

Cox Cure combined licensability classification with Cox survival modeling. For observed events ($s_{i}=1$):


$$\begin{array}{c} L_{\text{event}}=p_{i}\times h(t_{i}|X_{i})\times S(t_{i}|X_{i}) \end{array}$$ (5)

where $h(t_{i}|X_{i})=h_{0}(t_{i})\times \exp(\beta^{\prime }X_{i})$. For censored observations ($s_{i}=0$):


$$\begin{array}{c} L_{\text{censored}}=(1-p_{i})+p_{i}\times S(t_{i}|X_{i}) \end{array}$$ (6)

The total log-likelihood is:


$$\begin{array}{c} {ℒ}_{\text{cure}}=\sum_{i=1}^{n}\left[s_{i}\log L_{\text{event},i}+(1-s_{i})\log L_{\text{censored},i}\right] \end{array}$$ (7)

The cumulative baseline hazard $H_{0}(t)=\int_{0}^{t}h_{0}(u)du$ was computed via trapezoidal rule with 20 grid points during training and 50 during validation.

Classical Cox baseline optimized the standard Cox partial likelihood using hand-crafted features as implemented in scikit-survival [30].

### Experimental setup

We evaluated out-of-sample performance on the test set (2016–2017) using four modeling approaches to address fundamental questions about patent licensing prediction. The comparison evaluates whether deep learning provides value beyond classical approaches, whether temporal modeling improves over static classification, and whether explicit cure rate modeling enhances performance when many patents may never be licensed.

Our evaluation employed Recall@K as the primary metric, measuring the proportion of actual licensing events captured within the top K% of patents ranked by predicted cumulative licensing probability. We evaluated Recall@K at 1, 3, and 5 years post-publication with retrieval thresholds of 1%, 5%, 10%, 20%, and 30% to provide comprehensive coverage of practical use cases. Supplementary metrics included Lift@K (ratio of precision to base rate, quantifying business value) and concordance index (C-index, measuring rank-ordering capability for survival models).

We focus on recall-based metrics rather than precision-based measures because patent licensing data in the USPTO Patent Assignment Dataset reflects voluntary disclosures rather than mandatory reporting requirements. Patents labeled as unlicensed may include cases where licensing occurred but was not disclosed or captured in available records, creating false negatives that make precision calculations unreliable. Recall-based metrics remain valid as they measure the proportion of known licensing events successfully identified, providing actionable guidance for patent portfolio screening despite incomplete reporting.

For patents with multiple recorded licensing events, we used the earliest license date as the survival event time, treating the first observed licensing transaction as the primary event of commercial interest.

Statistical significance of performance differences was assessed using patent-level bootstrap resampling (*B* = 1,000 resamples, seed = 42). For each resample, a bootstrap sample of *n* = 624,129 patents was drawn with replacement from the test set using a single index shared across all models, ensuring paired comparisons. Within each resample, Recall@10% was computed for every model at the 1-, 3-, and 5-year horizons, and each pairwise model difference was averaged across the three horizons to yield one horizon-averaged difference per resample; the resulting *B* horizon-averaged differences form the bootstrap distribution for each comparison. Two-sided bootstrap *p*-values were computed by recentring this distribution at zero under the null hypothesis and measuring the proportion of recentred values at least as extreme as the observed mean difference, bounded below by the resolution 1/*B* = 0.001. Ninety-five percent confidence intervals were obtained by the percentile method.

## Results

We compared four modeling approaches—classical Cox regression, deep neural network baseline, DeepSurv, and Cox cure rate model on patent licensing prediction across three time horizons (1-year, 3-year, and 5-year). Our evaluation focused on Recall@K as the primary metric, measuring the proportion of licensing events captured at various retrieval thresholds, supplemented by Lift@K and concordance index (C-index).

**Fig 3:**
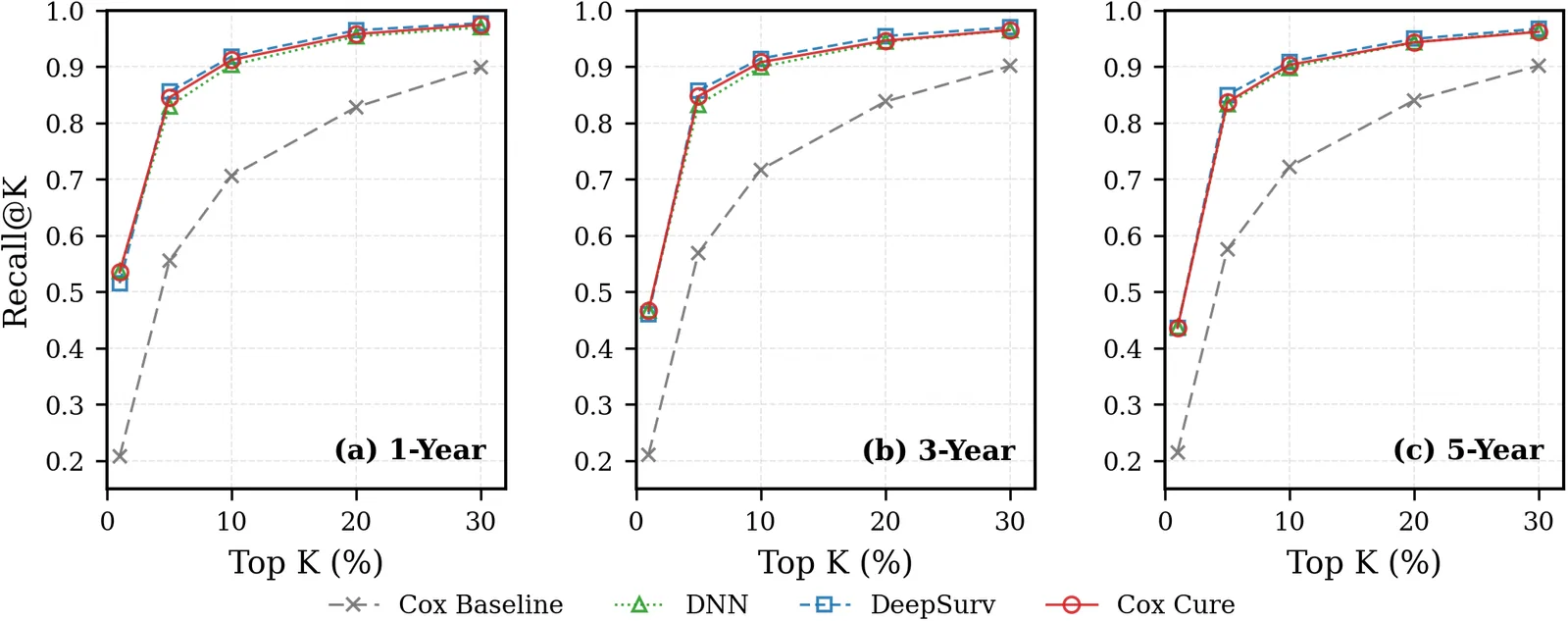
Recall@K performance comparison across four models and three prediction horizons. (a) 1-year prediction, (b) 3-year prediction, (c) 5-year prediction. Curves are distinguished by both line style and marker: Cox Baseline (gray, long-dashed, cross markers), DNN (green, dotted, open triangles), DeepSurv (blue, dashed, open squares), and Cox Cure (red, solid, open circles). Open markers allow overlapping points among the three deep models to remain individually identifiable.

All deep learning approaches demonstrated substantial improvements over classical Cox regression (Fig 3). At the critical 10% threshold, where practical patent screening typically operates, deep learning models achieved Recall@10% of 90.3–91.9% for 1-year predictions, 89.9–91.5% for 3-year predictions, and 89.8–90.9% for 5-year predictions. In contrast, classical Cox baseline achieved only 70.6%, 71.7%, and 72.3% for the respective horizons—representing a performance gap of approximately 19 percentage points. This improvement was consistent across all retrieval thresholds. At the more selective 5% threshold, deep learning models captured 83.0–85.8% of licensing events across horizons, compared to 55.6–57.6% for classical Cox baseline.

Concordance index results corroborated these findings (Table 1). Neural survival models achieved C-index values above 0.95, substantially higher than classical Cox baseline (0.896), confirming superior rank-ordering capability throughout the entire risk distribution. The DNN baseline was not evaluated using C-index as it performs static binary classification without modeling survival distributions, making concordance-based ranking inappropriate for this approach.

**Table 1:** Concordance index (C-index) for survival models. Higher values indicate better discrimination between patents that will and will not be licensed, with proper accounting for censoring and timing.

Model	C-index
Classical Cox Baseline	0.8956
DeepSurv	0.9548
Cox Cure	0.9518

Despite the theoretical appeal of explicitly modeling population heterogeneity, Cox Cure models showed minimal performance advantage over standard DeepSurv. At Recall@10%, Cox Cure achieved 91.0%, 90.8%, and 90.3% across the three horizons, while DeepSurv achieved 91.9%, 91.5%, and 90.9%, respectively—differences of only 0.6–0.9 percentage points. Per-model point estimates and their 95% bootstrap confidence intervals at each horizon are reported in Table 2. Bootstrap resampling confirmed these observations (Table 3). DeepSurv achieved a small but statistically detectable advantage over Cox Cure (mean difference = 0.70 percentage points, 95% CI = [0.30, 1.13], *p* < 0.001). While this difference is statistically significant given the large sample size (*n* = 624,129), the absolute gap of less than one percentage point is negligible for practical patent screening purposes, indicating that the additional architectural complexity of cure rate modeling provides no meaningful predictive benefit in this setting. Both models substantially outperformed the classical Cox baseline (mean difference > 18 percentage points, 95% CI lower bound > 17 percentage points, *p* < 0.001).

**Table 2:** Recall@10% point estimates with 95% bootstrap confidence intervals (percentile method, *B* = 1,000 patent-level resamples, *n* = 624,129 patents) for each model at the 1-, 3-, and 5-year prediction horizons.

Model	1-Year	3-Year	5-Year
Cox Baseline	0.7064 [0.6918, 0.7200]	0.7171 [0.7064, 0.7278]	0.7226 [0.7128, 0.7333]
DNN	0.9030 [0.8935, 0.9123]	0.8994 [0.8912, 0.9068]	0.8985 [0.8912, 0.9059]
DeepSurv	0.9186 [0.9103, 0.9272]	0.9145 [0.9073, 0.9216]	0.9091 [0.9022, 0.9159]
Cox Cure	0.9101 [0.9011, 0.9193]	0.9080 [0.9008, 0.9151]	0.9032 [0.8964, 0.9100]

**Table 3:** Pairwise statistical comparisons of Recall@10% using patent-level bootstrap resampling (*B* = 1,000, *n* = 624,129 patents). For each resample, the pairwise difference was averaged across the 1-, 3-, and 5-year horizons; statistics are computed over the resulting *B* horizon-averaged differences. Mean difference and 95% CI are in percentage points (pp). *p*-values are two-sided bootstrap *p*-values (minimum resolvable value 1/*B* = 0.001). * *p* < 0.05.

Comparison	Mean Diff. (pp)	95% CI	*p*-value
DeepSurv vs Cox Baseline	+19.83*	[18.78, 20.94]	<0.001
Cox Cure vs Cox Baseline	+19.14*	[18.09, 20.22]	<0.001
DNN vs Cox Baseline	+18.45*	[17.37, 19.50]	<0.001
DeepSurv vs Cox Cure	+0.70*	[0.30, 1.13]	<0.001
DeepSurv vs DNN	+1.38*	[0.92, 1.84]	<0.001
Cox Cure vs DNN	+0.69*	[0.20, 1.16]	0.004

* *p* < 0.05

Survival models (DeepSurv and Cox Cure) demonstrated modest but statistically significant improvements over static DNN binary classification. DeepSurv achieved meaningful performance gains over DNN (mean difference = 1.38 percentage points, 95% CI = [0.92, 1.84], *p* < 0.001), with Recall@10% differences of 1.1–1.6 percentage points across prediction horizons. Cox Cure similarly outperformed DNN (mean difference = 0.69 percentage points, 95% CI = [0.20, 1.16], *p* = 0.004), confirming that proper temporal modeling and handling of right-censoring provides statistically detectable benefits over static classification.

All deep learning models demonstrated high concentration of licensing events in top-ranked patents. Lift@K metrics (S1 Fig) show that at 10% retrieval, models achieved Lift values of 9.0–9.2 across horizons—approximately 9 times better than random selection.

All models maintained consistent relative performance across the three prediction horizons. The rank ordering remained stable: DeepSurv performed best, followed closely by Cox Cure, then DNN, with classical Cox baseline trailing substantially. Base rates increased from 0.60% (1-year) to 0.96% (3-year) to 1.11% (5-year), reflecting the cumulative nature of licensing events, yet relative model performance remained remarkably consistent.

## Discussion

The substantial performance gap between classical and deep approaches (Recall@10% differences exceeding 18 percentage points) versus modest differences among neural variants (below 1.5 percentage points) indicates that the primary modeling challenge in patent licensing prediction lies in capturing complex nonlinear relationships in patent characteristics, not in explicitly modeling population heterogeneity or cure fractions. Within this dataset and experimental setting, performance gains appeared to stem primarily from deep feature learning rather than from explicit population heterogeneity modeling. The modest improvement of survival models over static DNN classification (Recall@10% differences of 0.5–1.6 percentage points) suggests that proper temporal modeling and handling of right-censoring provides statistically detectable but practically limited additional value beyond static classification approaches. As noted in the Model Architectures section, the classical-versus-deep gap reflects a difference between entire modeling pipelines rather than architectures alone, whereas the comparisons among the three deep models—which share an identical feature encoder—isolate the temporal and cure-modeling components; the small differences among the latter therefore implicate feature learning, rather than temporal or cure modeling, as the primary driver of performance.

Three factors may explain why cure rate modeling provided limited additional benefit in this particular setting. First, the extremely low observed licensing rate (1.26%), combined with voluntary USPTO disclosure, creates inherent ambiguity between truly unlicensable patents and those whose licensing events have not yet occurred or been reported. Second, standard DeepSurv may implicitly capture population heterogeneity through its learned representations, assigning very low hazard rates to unlikely-to-be-licensed patents without requiring explicit cure rate components. Third, the added architectural complexity of jointly optimizing licensability probability, Cox hazard ratios, and baseline hazard functions may introduce optimization challenges that offset theoretical advantages in this extreme class imbalance setting.

Prior work on patent licensing prediction has used machine learning for static classification [13,23,24,31] or survival analysis for causal inference [15]. Rhee et al. [17] recently applied DeepSurv to patent transaction timing on Korean brokered transaction data, demonstrating its advantages over classical survival models. Our study extends this foundation by addressing three unresolved questions. First, we systematically evaluate cure rate models against standard DeepSurv, finding that explicit population heterogeneity modeling provided no clear additional benefit under our experimental conditions when deep feature learning is employed. Second, we quantify the gains from temporal modeling over static DNN classification (Recall@10% differences of 0.5–1.6 percentage points), demonstrating that survival analysis provides measurable but modest improvements. Third, we establish these findings on large-scale USPTO data (624,129 patents), demonstrating practical viability despite voluntary disclosure limitations. The key finding—that architectural simplicity suffices when deep feature learning is prioritized—provides actionable guidance for patent portfolio management.

Our findings are subject to several limitations. Licensing data reflects voluntary USPTO disclosures, introducing false negatives from both unreported licensing transactions and events not yet observed within the observation window. This affects model training by potentially biasing toward patents with characteristics associated with disclosure propensity rather than pure licensing probability. However, this limitation affects all models equally, preserving our comparative conclusions. The text embeddings for all models were derived from BERT-for-Patents [29] (Google, 2020), whose pre-training corpus has no publicly documented temporal coverage; we therefore cannot rule out that texts from our 2016–2017 test period were seen during pre-training. Because the same embeddings feed all four models and we performed no fine-tuning, any such leakage is shared across models and may affect absolute performance figures but not the relative comparisons that constitute our primary contribution. Finally, the scope of our conclusions is deliberately bounded by our study design. Because we restricted the comparison to the continuous-time Cox-based family in order to enable a controlled, ablation-style analysis (see Methods), our findings speak to which components matter *within* this family, and do not extend to survival paradigms built on different foundations. Approaches such as Random Survival Forests [28] (tree-based, without a proportional-hazards assumption) and DeepHit [25] (discrete-time competing risks) may offer different performance–complexity trade-offs, and a broader comparison across modeling paradigms remains an important direction for future work. Future work could also explore international patent licensing data, incorporate additional temporal features, or develop methods to account for disclosure propensity in model training.

## Conclusion

This study provides, to the best of our knowledge, the first systematic evaluation of continuous-time, Cox-based deep survival analysis frameworks for large-scale patent licensing prediction. Our comparative analysis on USPTO patents demonstrates that deep learning approaches substantially outperform classical Cox regression, with all neural models achieving high performance (Recall@10% 89.8–91.9%). Critically, standard DeepSurv achieves near-optimal performance without requiring complex cure rate extensions, indicating that, within the present experimental scope, the Cox cure model did not yield clear additional benefit over standard DeepSurv, and gains appear to stem primarily from deep feature learning. For practical deployment, DeepSurv offers the optimal balance of simplicity, lower implementation complexity, and performance, enabling organizations to achieve >90% recall at 10% retrieval for cost-effective patent portfolio screening.

## Supporting information

S1 Fig Lift@K performance comparison across four models and three prediction horizons. (a) 1-year prediction, (b) 3-year prediction, (c) 5-year prediction. Curves are distinguished by both line style and marker: Cox Baseline (gray, long-dashed, cross markers), DNN (green, dotted, open triangles), DeepSurv (blue, dashed, open squares), and Cox Cure (red, solid, open circles). Lift quantifies business value as the ratio of precision at threshold K to base rate. All deep learning approaches achieve substantially higher lift than classical baseline, with Lift@10% values of 9.0–9.2 indicating that the top 10% of patents are approximately 9 times more likely to be licensed than random selection.
(TIFF)
